# A Trigeminal Neuropathy From an Inactive Hepatocellular Carcinoma

**DOI:** 10.7759/cureus.20340

**Published:** 2021-12-10

**Authors:** Ananth Ram, Reji Paul, Vineeth Viswam, Biji Aravind

**Affiliations:** 1 Neurology, Aster Medcity, Kochi, IND; 2 Otolaryngology - Head and Neck Surgery, Aster Medcity, Kochi, IND; 3 Pathology, Aster Medcity, Kochi, IND

**Keywords:** solitary bone metastasis, trigeminal neuropathy, trigeminal neuralgia, skull base metastasis, hepatocellular carcinoma

## Abstract

Breast, lung, prostate, thyroid, and kidney carcinomas are the primary tumors that are known to have bony metastasis. Hepatocellular carcinoma (HCC) frequently involves the lung and lymph nodes and less commonly the osseous system. Numbness/persistent pain in the distribution of the trigeminal nerve is more likely a neuropathy. The causes are idiopathic(common), unintentional injury to the trigeminal nerve during surgery or trauma, blood vessel pressing the trigeminal nerve, tumor infiltration, multiple sclerosis, and stroke. Unresolved facial pains after conventional treatment should prompt additional investigation to rule out other causes. In this case, we report a trigeminal neuropathy of rare cause, which is a solitary metastasis from an inactive HCC involving the osseous structures.

## Introduction

Breast, lung, prostate, thyroid, and kidney carcinomas are the primary tumors that are known to have bony metastasis [1]. Hepatocellular carcinoma (HCC) frequently involves the lung and lymph nodes and less commonly the osseous system. Numbness/persistent pain in the distribution of the trigeminal nerve is more likely a neuropathy. The causes are idiopathic (common), unintentional injury to the trigeminal nerve during surgery or trauma, blood vessel pressing the trigeminal nerve, tumor infiltration, multiple sclerosis, and stroke. Unresolved facial pains after conventional treatment should prompt additional investigation to rule out other causes.

## Case presentation

A 69-year-old lady, who is on treatment for diabetes, hypothyroidism, and hypertension, was detected to have HCC three years ago, a solitary space-occupying lesion in segment six of the liver. The patient underwent successful transarterial chemoembolization (TACE) and was cancer-free for the next three years.

The patient presented with a 10-day duration of right hemifacial pain that was of electric shock-like quality occurring frequently and precipitated by chewing food, wind blowing, and water splashing on the cheek, characteristic of trigeminal neuralgia. Cranial nerve examination at that time was normal. She was started on oxcarbazepine 300mg twice daily and the non-contrast MRI of the Brain was reported to be normal. Two weeks later, she presented with worsening hemifacial pain and doubling of vision. She described the pain as burning, associated with tinnitus, numbness of the right maxillary and mandibular divisions of the trigeminal nerve. Clinical examination revealed a right lateral rectus palsy, absent corneal reflex, and reduced pin sensation of the V2-V3 divisions of the right trigeminal nerve. The remainder of her neurological examination was normal.

**Figure 1:**
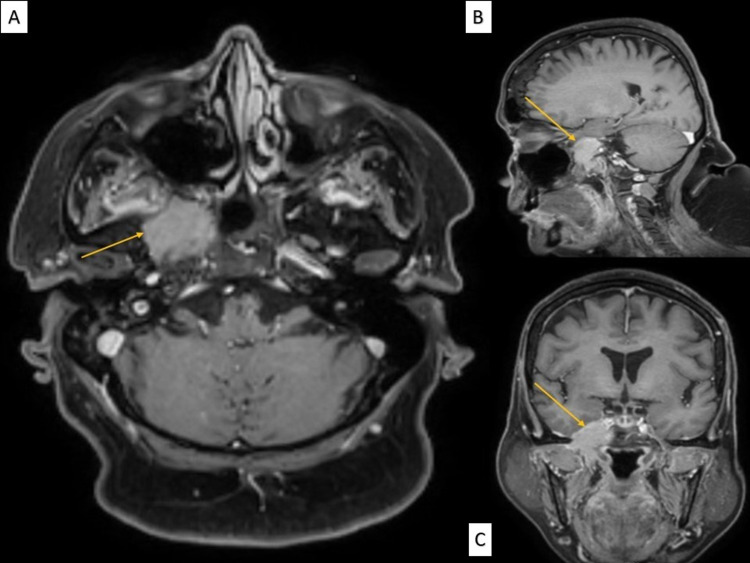
(A) Axial view, (B) sagittal view, and (C) coronal view of the MRI of the patient demonstrating a destructive solid heterogeneously enhancing mass of 2.9x2.4 cm (yellow arrow) centered in the medial portion of the right middle skull base involving the greater wing of sphenoid, lateral sphenoid body, and pterygoid plates extending into the intracranial portion and infratemporal fossa.

Given the above findings, an alternate diagnosis was suspected. A contrast MRI brain (Figures 1A-1C) revealed a 2.9x2.4 cm destructive solid heterogeneously enhancing mass centered in the medial portion of the right middle skull base involving the greater wing of sphenoid, lateral sphenoid body, and pterygoid plates extending into the intracranial portion and infratemporal fossa. There was bony erosion of the petrous segment and cavernous portion of the carotid canal with narrowing of the petrous and posterior cavernous portion of the right internal carotid artery and invasion of the right lateral sphenoid body and petrous apex involving the right abducens nerve.

**Figure 2:**
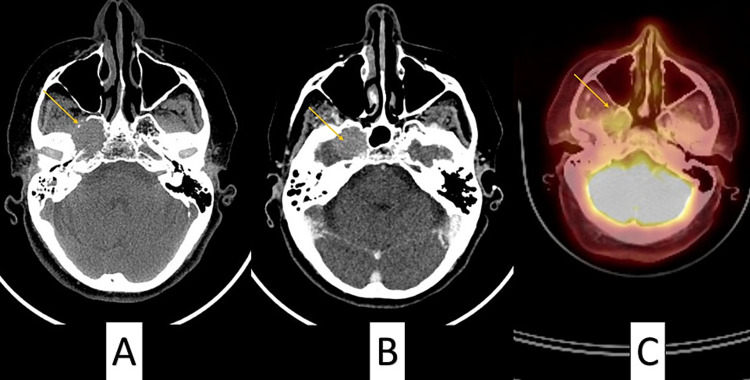
(A, B) Destructive solid heterogeneously enhancing mass of 2.9x2.4 cm (yellow arrow) centered in the medial portion of the right middle skull base involving greater wing of sphenoid, lateral sphenoid body, and pterygoid plates causing bony erosion of petrous segment. (C) PET CT showing mildly enhancing right skull base lesion in the same area as the location of the mass.

Diagnostic considerations included metastasis, primary aggressive lesion/ malignancy involving the middle skull base. Hence she underwent whole-body PET CT (Figures 2A-2C), which showed mildly enhancing right skull base lesion as noted in the MRI, which appears to wash off in the delayed sequences, suggesting a possibility of inflammatory vs neoplastic lesion. No FDG avid suspicious lesions were noted at the post-TACE site in segment six of the liver or the remaining liver parenchyma.

The patient underwent an endoscopic skull base biopsy. Via a transnasal approach, the mass of 2.9x2.4x3.4 cm was located just lateral to the paraclival and petrous portion of the carotid artery. The entire mass and a small fungal ball in the posterior ethmoid sinus were removed.

**Figure 3:**
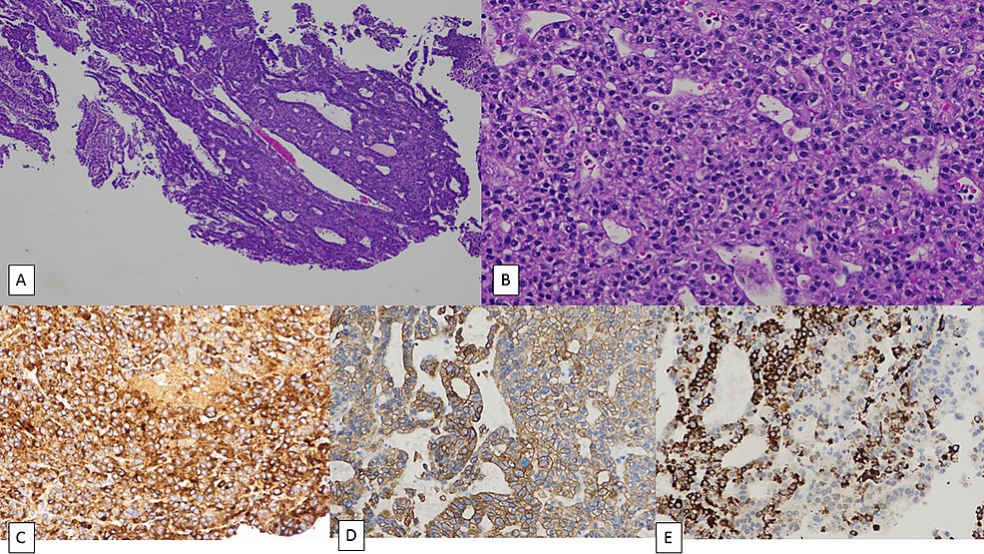
(A) Fragments of neoplasm with cells arranged as cords and trabeculae, H&E x 40. (B) The neoplastic cells show eosinophilic cytoplasm and nuclei with mild pleomorphism, H&E x 200. (C) IHC for glypican showing strong diffuse positivity x 200. (D) IHC for cytokeratin showing diffuse positivity x 200. (E) IHC for hepar showing patchy positivity x 200. IHC - Immunohistochemistry

The histopathological analysis showed fragments of a neoplasm composed of trabeculae and glands lined by cells with eosinophilic nuclei and pleomorphic nuclei with nucleoli and occasional inclusions. Immunohistochemistry analysis (Figures 3A-3E) showed strong positivity for glycipan 3 and patchy positivity for hepar and cytokeratin and negative for CK7, CK20, GATA 3, PAX8, TTF1, S100, and EMA suggesting metastasis from HCC.

The patient improved symptomatically and is currently symptom-free.

## Discussion

According to the International Classification of Headache Disorders, typical trigeminal neuralgia is characterized by recurrent paroxysms of unilateral pain in the distribution of one or more divisions of the trigeminal nerve, which does not radiate beyond the distribution, lasts for seconds to two minutes, the severe intensity with an electric shock-like quality and precipitated by innocuous stimuli [2]. This was consistent with our patient. Our patient had involvement of V2-3 divisions of the right trigeminal nerve and right sixth nerve.

The differential diagnosis to consider in the setting of multiple cranial nerve palsy in the background of HCC are meningeal carcinomatosis, meningeal infections, and skull base metastasis, although radiological and spinal fluid analysis would help in differentiating most cases.

Incidence of HCC metastasis accounts for about 5%, most often involving the lungs and the regional lymph nodes [3]. Involvement of the osseous structures is seen in about 2% to 16%, which are the vertebrae, ribs, pelvic, and long bones [4]. Metastasis to the skull base is reported to be 0.4%-1.6%. Solitary metastasis to the skull base without symptomatic evidence of primary in the liver or other sites is an extremely rare and uncommon presentation of HCC [5].

Hsieh et al. [6] found 68 patients with skull metastasis from HCC. The most common presentation was a subcutaneous liver mass with occasional painful sensations, followed by cranial nerve deficits, such as dysphagia, deafness, visual disturbances, facial numbness, of which the latter two symptoms were noted in our patient. Rad et al. reported a solitary cavernous sinus metastasis from HCC without metastasis to other sites in a patient who presented with acute painful ophthalmoplegia. Similar presentations have been reported by Goto et al. [7] and Kato et al. [8].

Trivedi et al. [9] reported a patient with an isolated skull base metastasis as the first manifestation of HCC, who presented with right 3,4,6 cranial nerve palsy and upon evaluation was noted to have HCC.

## Conclusions

The uniqueness about our patient is that she had a cancer-free liver and presented with symptoms suggestive of trigeminal neuropathy. Our patient neither had a liver dysfunction at presentation nor at any time during her admission.

Unresolved trigeminal neuralgia despite medical management, needs detailed evaluation and skull base metastasis from tumors, isolated or not, should be ruled out. Early investigation and management improve patients' quality of life.
